# Resolving a taxonomic and nomenclatural puzzle in mantellid frogs: synonymization of *Gephyromantis azzurrae* with *G. corvus*, and description of *Gephyromantis kintana* sp. nov. from the Isalo Massif, western Madagascar

**DOI:** 10.3897/zookeys.951.51129

**Published:** 2020-07-22

**Authors:** Walter Cocca, Franco Andreone, Francesco Belluardo, Gonçalo M. Rosa, Jasmin E. Randrianirina, Frank Glaw, Angelica Crottini

**Affiliations:** 1 CIBIO, Research Centre in Biodiversity and Genetic Resources, InBIO, Universidade do Porto, Campus Agrário de Vairão, Rua Padre Armando Quintas, No 7, 4485-661 Vairão, Portugal; 2 Sezione di Zoologia, Museo Regionale di Scienze Naturali, Via G. Giolitti, 36, 10123 Torino, Italy; 3 Institute of Zoology, Zoological Society of London, Regent’s Park, NW1 4RY London, UK; 4 Centre for Ecology, Evolution and Environmental Changes (cE3c), Faculdade de Ciências da Universidade de Lisboa, Bloco C2, Campo Grande, 1749-016 Lisboa, Portugal; 5 Parc Botanique et Zoologique de Tsimbazaza, BP 4096, Antananarivo 101, Madagascar; 6 Zoologische Staatssammlung München (ZSM-SNSB), Münchhausenstraße 21, 81247 München, Germany; 7 Departamento de Biologia, Faculdade de Ciências da Universidade do Porto, R. Campo Alegre, s/n, 4169-007, Porto, Portugal

**Keywords:** Amphibia, Mantellidae, Mantellinae, *
Phylacomantis
*, integrative taxonomy

## Abstract

The genus *Gephyromantis* belongs to the species-rich family Mantellidae and is currently divided in six subgenera. Among these is the subgenus Phylacomantis, which currently includes four described species: *Gephyromantis pseudoasper*, *G. corvus*, *G. azzurrae*, and *G. atsingy*. The latter three species are distributed in western Madagascar, and two of them (*G. azzurrae* and *G. corvus*) occur in the Isalo Massif. Based on the analysis of molecular data (a fragment of the 16S rRNA gene), morphological inspection of museum specimens, and photographic comparisons, *G. azzurrae* is synonymised with *G. corvus* and the second *Phylacomantis* lineage of Isalo is described as *G. kintana***sp. nov.** This medium-sized frog species (adult snout-vent length 35–44 mm) is assigned to this subgenus according to genetic and morphological similarities to the other known species of *Phylacomantis*. *Gephyromantis kintana***sp. nov.** is known only from the Isalo Massif, while new records for *G. corvus* extend its range to ca. 200 km off its currently known distribution. These two taxa seem to occur in syntopy in at least one locality in Isalo, and the easiest way to distinguish them is the inspection of the ventral colouration, dark in *G. corvus* and dirty white in *G. kintana*.

## Introduction

The biodiversity hotspot of Madagascar hosts a unique, diverse, and imperilled ecosystem (Myers et al. 2000; Goodman and Benstead 2003, 2005). The island’s amphibians contribute significantly to its rich biodiversity with 100% of the autochthonous species being endemic to the country (Glaw and Vences 2007; Perl et al. 2014; Zimkus et al. 2017). All native amphibians of Madagascar are anurans and belong to four distinct families: Mantellidae Laurent, 1946, Microhylidae Günther, 1858, Hyperoliidae Laurent, 1943 and Ptychadenidae Dubois, 1987 (Glaw and Vences 2007; Crottini et al. 2012). The family Mantellidae is the most species rich clade with ca. 230 currently described species (AmphibiaWeb 2020) and several new species are awaiting formal description (Vieites et al. 2009; Perl et al. 2014). Mantellids are divided in three subfamilies, the Boophinae Vences & Glaw, 2001 (with 79 described species), the Laliostominae Vences & Glaw, 2001 (with seven described species), and the Mantellinae Laurent, 1946 (with 143 described species) (AmphibiaWeb 2020). Based on significant genetic differentiation, habitat requirement and morphology mantellin frogs are classified in nine recognised genera: *Blommersia* Dubois, 1992, *Boehmantis* Glaw & Vences, 2006, *Gephyromantis* Methuen, 1920, *Guibemantis* Dubois, 1992, *Mantella* Boulenger, 1882, *Mantidactylus* Boulenger, 1895, *Spinomantis* Dubois, 1992, *Tsingymantis* Glaw, Hoegg and Vences 2006 and *Wakea* Glaw & Vences, 2006 (Glaw and Vences 2006, 2007).

The genus *Gephyromantis* is currently divided in six subgenera: *Gephyromantis* Methuen, 1920, *Laurentomantis* Dubois, 1980, *Vatomantis* Glaw & Vences, 2006, *Phylacomantis* Glaw & Vences, 1994, *Duboimantis* Glaw & Vences, 2006 and *Asperomantis* Vences, Köhler, Pabijan, Bletz, Gehring, Hawlitschek, Rakotoarison, Ratsoavina, Andreone, Crottini & Glaw, 2017.

*Gephyromantis* are mostly small to medium-sized frogs that, for a long time, most of them were thought to be direct developers (not depending on water bodies for their larval development). However, and despite development being unknown for the majority of the species, free-swimming, exotrophic tadpoles have been recorded in some of them (Glaw and Vences 2007; Randrianiaina et al. 2011). In addition, endotrophic (non-feeding) nidicolous tadpoles, genetically identified as belonging to the subgenus Duboimantis, were recently identified (Randrianiaina et al. 2011). Eggs of these species are most probably laid into the leaf-litter and washed into streams where they complete the larval development and metamorphosis (Randrianiaina et al. 2011). The majority of the *Gephyromantis* species can be found in the low and mid-altitude rainforest of the north and east of Madagascar, with the exception of most species of the subgenus Phylacomantis, which primarily occupy western Madagascar (Glaw and Vences 2007; Mercurio and Andreone 2007; Crottini et al. 2011a; Andreone et al. 2014; Cocca et al. 2018).

The subgenus Phylacomantis currently contains four described species distributed in the north, west and south-west of Madagascar: *G. pseudoasper* (Guibé, 1974), *G. corvus* (Glaw & Vences, 1994), *G. azzurrae* Mercurio & Andreone, 2007, and *G. atsingy* Crottini, Glaw, Casiraghi, Jenkins, Mercurio, Randrianantoandro, Randrianirina & Andreone, 2011. These medium-sized frogs are mostly terrestrial, being active mainly in crepuscular and night hours (Glaw and Vences 1994, 2006). With the exception of *G. pseudoasper*, which can be found far from water bodies, all the species are typically encountered in rocky habitats along small streams in dry deciduous forest (Glaw and Vences 2006, 2007). Males are often heard calling from the ground, from bushes or trees at relatively low perch. Some species of the subgenus Phylacomantis are known to have exotrophic carnivorous tadpoles capable of emitting sounds, possibly as an aggressive signal towards conspecific tadpoles during prey capture (Reeve et al. 2011).

In this paper we combined available evidence (morphological and genetic data, photographic material) on the two *Phylacomantis* species inhabiting the Isalo Massif (currently referred to as *G. azzurrae* and *G. corvus*) and compared it with recently collected material. The results of this analysis point to the need to synonymise the name *Gephyromantis azzurrae* with *G. corvus* and describe a new taxon that had for long time remained hidden in plain sight (i.e., under the name *G. corvus*).

## Materials and methods

### Study sites

**Figure 1.**
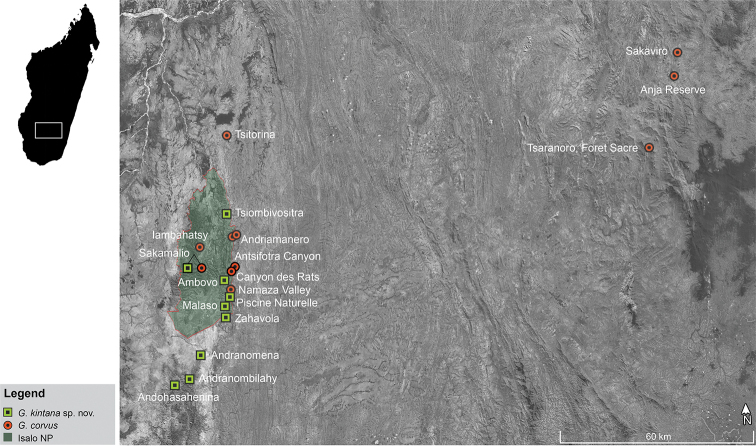
Distribution of *Gephyromantis corvus* and *G. kintana* sp. nov. Note that in Sakamalio the two species are found in syntopy.

The Isalo Massif is situated in the southwestern corner of the Ihorombe region. A large portion of the massif is included within the Parc National de l’Isalo, one of the largest protected areas in Madagascar (81,540 ha). It consists of a low to mid-altitude mountain range (altitudinal range from 500 to 1,300 m a.s.l.), characterised by the occurrence of numerous canyons and valleys, varying in size, depth and in the level of humidity and water availability. This area hosts numerous patches of dry deciduous forest, which are generally associated to streams within the canyon system (Fig. 1; Table 1; Mercurio and Andreone 2007; Mercurio et al. 2008; Cocca et al. 2018).

In addition to Isalo, we surveyed an area close to the Andringitra Massif (in the south-east): we found individuals of *Phylacomantis* in Tsaranoro forest, Anja Reserve and Sakaviro Community Reserve, all within the administrative region of Haute Matsiatra (Fig. 1). Tsaranoro forest is a small fragment of ca. 46 ha, ca. 4 km away from the western entrance of the Parc National d’Andringitra and it is characterised by a semi-deciduous dry forest surrounded by villages and rice fields (Fig. 1; Gould and Andrianomena 2015). Anja Reserve is located ca. 13 km south of Ambalavao, and is characterised by the presence of forest fragments at the base of some large granitic boulders known to host several microendemic species (Crottini et al. 2011b, 2012, 2015). Sakaviro Community Reserve is a small (ca. 14 ha) remaining fragment of semi-deciduous dry forest at the base of a granitic dome, which is located ca. 8 km north of Anja Reserve (Fig. 1; Table 1).

**Table 1.** List of toponyms and corresponding GPS coordinates and altitudes.

Locality	Latitude / Longitude	Altitude [m a.s.l.]
Ambovo	-22.50800000S, 45.35250000E	999
Andohasahenina	-22.83333300S, 45.18800000E	876
Andranombilahy	-22.55000000S, 45.41670000E	920
Andranomena	-22.74016700S, 45.27500000E	740
Andriamanero 1	-22.36716700S, 45.39200000E	663
Andriamanero 2	-22.37333333S, 45.37850000E	792
Anja	-21.85962000S, 46.85827000E	970
Antsifotra Canyon	-22.42120000S, 45.27450000E	743
Canyon des Rats	-22.47987500S, 45.37663200E	841
Iambahatsy	-22.40583300S, 45.26883300E	742
Malaso	-22.58850000S, 45.35533333E	966
Namazaha Valley	-22.55000000S, 45.41670000E	820
Piscine Naturelle	-22.55966700S, 45.37183300E	841
Sakamalio	-22.43483300S, 45.25516700E	726
Sakaviro	-22.42120000S, 45.27450000E	1018
Tsaranoro	-22.08473000S, 46.77515000E	946
Tsiombivositra	-22.30250000S, 45.35833300E	900
Tsitorina	-22.05816700S, 45.35616700E	465
Zahavola	-22.62153610S, 45.35866700E	881

### Voucher collection

**Figure 2.**
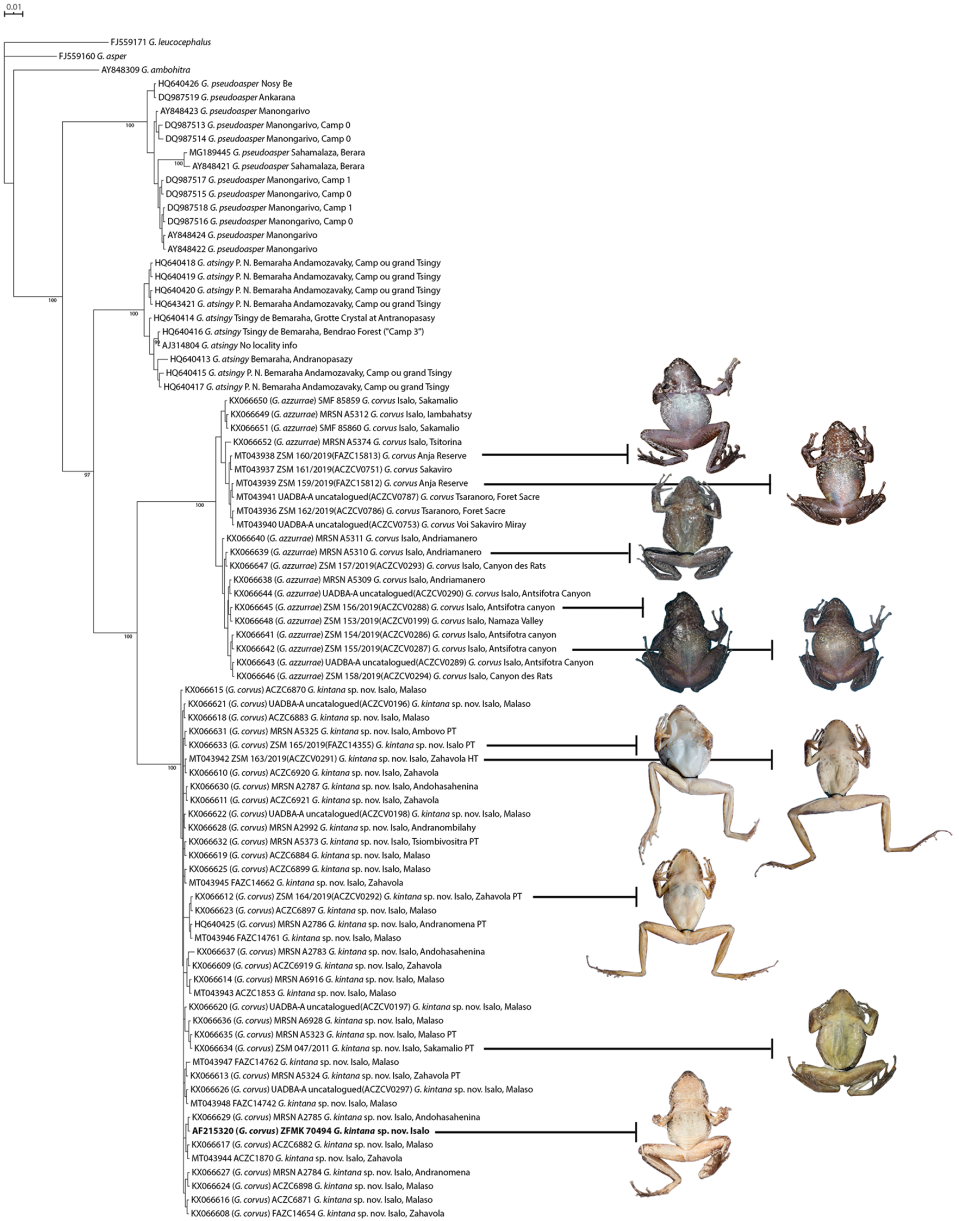
Phylogenetic consensus tree of the subgenus Phylacomantis. Bayesian majority rule consensus tree based on a fragment of the mitochondrial 16S rRNA gene. Numbers at nodes are Posterior Probability (PP) values. In bold is highlighted the sequence (AF215320) of the adult male (ZFMK 70494) that was used as genetic reference for *G. corvus* in Vences (2000) and following contributions. Each sequence is reported with the following information: GenBank accession number, GenBank taxon identification (given in parenthesis when taxon ID does not match with the currently proposed definition), institutional catalogue number and/or field number (when specimen was not collected), species ID, locality. *G. leucocephalus*, *G. asper*, and *G. ambohitra* were used as outgroups.

Frogs were searched during the day and night (using headlamps and torches). The position of each site was recorded with a GPS device. Special efforts have been invested in collecting specimens at Namazaha (or Namaza) Valley, the type locality of *G. corvus* within the Isalo N.P. Twenty individuals (collected over several years) were euthanised by immersion in a solution of MS-222, fixed in 96% ethanol and stored in 70% ethanol. From each voucher specimen we collected a tissue sample, which was preserved separately in 96% ethanol for genetic analyses. Vouchers were deposited in the herpetological collection of the Zoologische Staatssammlung München, Germany (**ZSM**), and of the Mention “Zoologie et Biodiversité Animale” of the University of Antananarivo, Madagascar (**UADBA-A**) (for detailed information on the collections material please refer to Fig. 2). The eight vouchers hosted in the UADBA-A collection were analysed genetically but have not been measured. Other institutional abbreviations used herein are

**MRSN** Museo Regionale di Scienze Naturali di Torino, Italy

**ZFMK** Zoologisches Forschungsmuseum Alexander Koenig, Bonn, Germany

**SMF**Senckenberg Museum Frankfurt, Germany.

Codes ACZC and ACZCV refer to field numbers of A. Crottini and the code FAZC refers to field numbers of F. Andreone.

### Morphological measurements

Morphological measurements (in mm) were taken with a digital calliper to the nearest 0.1 mm by W. Cocca (Table 2):

**ED** horizontal eye diameter,

**END** eye-nostril distance, measured from the anterior corner of eye to the centre of the nostril,

**FORL** forelimb length, measured from the axilla to the tip of the longest (third) finger with the forelimb extended,

**FOTL** foot length including tarsus, measured from the tibio-tarsal articulation to the tip of the longest (fourth) toe,

**HAL** hand length, measured from the base of the hand to the tip of the longest (third) finger,

**HIL** hind-limb length, measured from the cloaca to the tip of the longest (fourth) toe with the foot extended laterally outward from the body,

**HIL/SVL** ratio between hind-limb length and snout-vent length,

**HL** head length, measured as the diagonal from the maxillary commissure to the snout tip (Note: this is measured along the jaw, and not parallel to the longitudinal axis of the animal),

**HW** head width at widest point,

**IMTL** length of inner metatarsal tubercle,

**NND** nostril-nostril distance, measured from the centre of the nostrils,

**NSD** nostril-snout tip distance, measured from the centre of the nostril,

**SVL** snout-vent length,

**TD** horizontal tympanum diameter,

**TD/ED** ratio between horizontal tympanum diameter and horizontal eye diameter,

**TIBL** point reached by tibio-tarsal articulation when hindlimbs are adpressed along body).

For adult male individuals we also collected measurements for the femoral macrogland cluster:

**FGL** length of the femoral macrogland cluster,

**FGW** width of femoral macrogland cluster,

**GD** mean diameter of granules composing the right femoral gland,

**NG** number of granules composing the right femoral gland.

Granules were counted after having opened and flipped the skin where the gland is located. Webbing formula follows Blommers-Schlösser (1979), and femoral glands definition follows Glaw et al. (2000). Terminology and description scheme follow Mercurio and Andreone (2007). Description of colour in life is based on the holotype, with some reference to variation as observed in paratypes.

### Molecular analysis

Total genomic DNA was extracted from tissue samples using proteinase K digestion (10 mg/ml concentration) followed by a standard high-salt extraction method (Bruford et al. 1992). We amplified a fragment of ca. 550 bp of the 3’ terminus of the mitochondrial 16S rRNA gene (hereafter 16S) proven to be suitable for amphibian identification (Vences et al. 2005; Vieites et al. 2009). We used the primers 16S-AR 5’-CGCCTGTTTATCAAAAACAT-3’ and 16S-BR 5’-CCGGTYTGAACTCAGATCAYGT-3’, modified from Palumbi et al. (1991), as described in Crottini et al. (2011a). Standard polymerase chain reactions (PCR) were performed in a final volume of 25 μL and using 0.75 μL each of 10 pmol primer, 0.4 μL of total dNTP 10 mM (Promega), 0.1 μL of 5 U/mL GoTaq, 5 μL 5X Green GoTaq Reaction Buffer (Promega) and 4 μl of MgCl_2_ 25mM (Promega). Successfully amplified and purified fragments were sequenced using dye-labelled dideoxy terminator cycle sequencing on an ABI 3730XL automated sequencer at Macrogen Inc. Chromatograms were checked and sequences were manually edited, where necessary, using the sequence alignment editor of BIOEDIT (v.7.2.0; Hall 1999). All new sequences have been deposited in GenBank (MT043936–MT043948; Table 2).

**Table 2.** Morphometric measurements of specimens of *Gephyromantis corvus* and *G. kintana* sp. nov. All measurements are in mm. Numbers in TIBL indicate different states: 1, eye, 2, nostril, 3, snout, 4, beyond the snout. HT (holotype), PT (paratype), M (male), F (female), * (subadult), Juv (juvenile), NA (not available), £ (type specimens of *G. azzurrae*).

Species	GenBank	Locality	Voucher	Fieldnumber	Type	Sex	SVL	HW	HL	ED	END	NSD	NND	TD	TD/ED	HAL	FORL	FOTL	IMTL	HIL	HIL/SVL	TIBL	NG	FGL	FGW	GD
*G. corvus*	Unavailable	Namazaha Valley	ZFMK 57430		HT	M	37.8	14.0	14.2	5.5	NA	NA	NA	2.7	0.5	11.5	38.4	25.0	1.1	56.0	1.5	1	38	8.1	2.7	0.2
*G. corvus*	Unavailable	Namazaha Valley	ZSM 574/1999 (ZFMK57431)		PT	M	37.0	13.5	13.8	4.2	NA	NA	NA	3.1	0.7	11.9	23.3	27.7	0.9	60.3	1.6	3	32	6.4	2.5	0.4
*G. corvus*	KX066639, EF222301	Andriamanero	MRSN A5310	FAZC12568	HT£	M	41.1	16.9	13.4	6.1	3.9	2.5	4.0	4.0	0.7	12.1	20.0	30.0	1.1	41.1	1.0	1	45	6.3	2.0	0.5
*G. corvus*	KX066638, EF222300	Andriamanero	MRSN A5309	FAZC12567	PT£	M	38.5	15.3	12.8	5.2	4.3	2.2	3.7	3.7	0.7	11.1	19.9	26.7	1.3	41.1	1.1	1	38	6.5	2.7	0.5
*G. corvus*	KX066640, EF222302	Andriamanero	MRSN A5311	FAZC12569	PT£	M	40.2	15.8	14.1	6.0	4.0	2.7	4.0	4.1	0.7	11.2	19.9	27.7	1.1	41.0	1.0	1	40	6.7	2.7	0.6
*G. corvus*	KX066650, EF222305	Sakamalio	SMF 85859 (MRSN A5314)	FAZC 12979	PT£	M	42.7	16.4	14.3	5.4	3.7	2.6	3.9	3.5	0.6	13.4	21.0	29.9	1.1	41.1	1.0	1	42	7.0	3.0	0.6
*G. corvus*	KX066651, EF222303	Sakamalio	SMF 85860 (MRSN A5315)	FAZC 12980	PT£	M	43.7	16.4	13.5	5.7	4.0	2.5	3.8	4.0	0.7	12.2	21.1	27.7	1.0	42.3	1.0	1	42	7.5	2.7	0.5
*G. corvus*	KX066642	Antsifotra Canyon	ZSM 155/2019	ACZCV_287		M	39.6	14.7	15.4	4.7	3.8	2.0	3.9	3.6	0.8	11.3	27.1	26.9	0.5	66.4	1.7	2	29	8.2	2.9	0.8
*G. corvus*	KX066645	Antsifotra Canyon	ZSM 156/2019	ACZCV_288		M	41.1	16.0	18.5	5.4	3.9	2.6	3.6	3.6	0.7	11.1	25.1	29.8	0.8	70.4	1.7	1	58	9.2	2.7	0.5
*G. corvus*	KX066648	Namazaha Valley	ZSM 153/2019	ACZCV_199		M	39.2	15.4	14.1	4.2	3.9	2.0	3.4	3.4	0.8	12.7	26.6	27.5	0.9	65.5	1.7	1	NA	NA	NA	NA
*G. corvus*	MT043936	Tsaranoro	ZSM 162/2019	ACZCV_786		M	41.8	14.6	14.0	4.2	3.9	2.9	3.4	3.0	0.7	12.2	27.4	28.3	0.8	62.6	1.5	1	43	7.4	2.6	0.3
*G. corvus*	MT043937	Sakaviro	ZSM 161/2019	ACZCV_751		M	39.3	15.4	14.4	4.1	4.0	2.2	3.9	1.8	0.4	11.6	24.0	27.8	0.7	62.1	1.6	2	43	7.6	2.8	0.4
*G. corvus*	MT043938	Anja	ZSM 160/2019	FAZC15813		M	41.1	14.4	14.3	4.7	4.3	1.9	3.3	3.3	0.7	12.5	25.5	30.2	0.9	70.3	1.7	3	45	7.9	2.5	0.4
*G. corvus*	MT043939	Anja	ZSM 159/2019	FAZC15812		F	42.0	14.3	15.1	5.2	3.8	2.5	3.3	3.0	0.6	11.3	24.4	28.4	0.5	64.1	1.5	2	NA	NA	NA	NA
*G. corvus*	KX066641	Antsifotra Canyon	ZSM 154/2019	ACZCV_286		F	37.2	13.8	14.2	4.9	4.4	2.9	3.5	3.3	0.7	10.3	24.3	27.0	0.4	60.5	1.6	1	NA	NA	NA	NA
*G. corvus*	KX066649, JN664352, EF222304	Iambahatsy	MRSN A5312	FAZC12910	PT£	M*	23.3	8.8	8.8	4.1	2.8	1.4	2.2	2.5	0.6	8.8	11.1	17.7	0.5	24.5	1.1	1	NA	NA	NA	NA
*G. corvus*	KX066647	Canyon des Rats	ZSM 157/2019	ACZCV_293		M*	27.4	11.3	11.2	3.2	2.8	1.5	2.4	2.0	0.6	9.0	18.5	21.2	0.4	44.8	1.6	3	35	4.8	2.0	0.1
*G. corvus*	KX066646	Canyon des Rats	ZSM 158/2019	ACZCV_294		Juv	21.4	8.1	8.5	3.6	2.0	1.4	2.2	1.7	0.5	5.8	13.0	15.4	0.1	35.2	1.6	4	NA	NA	NA	NA
*G. kintana*	MT043942	Zahavola	ZSM 163/2019	ACZCV_291	HT	M	38.2	15.6	14.6	4.0	4.1	2.5	3.4	3.0	0.8	11.5	25.1	30.4	0.8	67.5	1.8	4	71	9.4	3.6	0.3
*G. kintana*	KX066634, JN664348	Sakamalio	ZSM 0047/2011 (MRSN A5313)	FAZC12951	PT£	M	37.2	14.9	12.3	5.5	4.1	2.3	3.5	3.3	0.6	11.1	15.6	26.6	0.9	40.0	1.1	1	29	8.2	2.2	0.6
*G. kintana*	Unavailable	Malaso	MRSN A5322	FAZC12627	PT	M	43.6	15.2	16.5	5.5	NA	NA	NA	3.6	0.7	11.9	26.7	30.2	0.7	69.0	1.6	3	24	7.5	3.3	0.6
*G. kintana*	KX066632, HQ640423	Tsiombivositra	MRSN A5373	FAZC12859	PT	M	39.8	16.1	15.3	5.8	4.8	2.9	4.3	3.7	0.6	11.8	16.3	28.6	1.4	38.7	1.0	2	96	9.1	4.1	0.6
*G. kintana*	Unavailable	Piscine Naturelle	ZSM 1553/2009	NA	PT	M	35.7	13.8	15.0	4.9	NA	NA	NA	2.5	0.5	11.6	20.2	26.8	0.8	59.6	1.7	1	35	8.9	3.0	0.4
*G. kintana*	KX066633	Malaso	ZSM 165/2019	FAZC14355	PT	M	41.9	15.9	15.9	4.7	4.5	2.2	3.5	3.2	0.7	12.8	25.6	31.1	0.8	70.1	1.7	2	75	10.5	4.3	0.6
*G. kintana*	KX066612	Zahavola	ZSM 164/2019	ACZCV_292	PT	F	38.0	14.1	15.1	4.6	3.9	2.6	3.0	2.9	0.6	11.0	22.9	28.5	0.7	64.3	1.7	1	NA	NA	NA	NA
*G. kintana*	KX066613, HQ640424	Zahavola	MRSN A5324	FAZC12758	PT	F	40.1	15.0	16.3	6.1	4.4	2.8	4.2	3.3	0.5	10.8	16.9	28.0	0.7	37.2	0.9	2	NA	NA	NA	NA
*G. kintana*	KX066631	Ambovo	MRSN A5325	FAZC13000	PT	F	40.0	15.2	16.2	6.4	4.3	2.6	4.1	3.2	0.5	10.7	18.2	30.1	0.6	40.3	1.0	2	NA	NA	NA	NA
*G. kintana*	Unavailable	Ambovo	MRSN A5326	FAZC13001	PT	F	38.0	13.0	14.9	4.6	NA	NA	NA	2.9	0.6	9.6	23.1	27.9	0.7	62.6	1.6	3	NA	NA	NA	NA
*G. kintana*	HQ640425	Andranomena	MRSN A2786	FAZC11964	PT	F	40.8	15.0	15.8	6.3	4.7	2.7	4.3	3.6	0.6	11.1	17.7	30.2	0.7	40.5	1.0	2	NA	NA	NA	NA
*G. kintana*	KX066635, HQ640422	Malaso	MRSN A5323	FAZC12661	PT	F	39.0	15.0	15.7	6.1	4.4	2.8	4.2	3.6	0.6	11.9	17.6	29.7	0.8	39.3	1.0	2	NA	NA	NA	NA

We aligned the newly generated 16S sequences with all available homologous sequences of the species of the subgenus Phylacomantis (see Fig. 2 for details of GenBank accession numbers), and one sequence each of *G. ambohitra* (AY848309), *G. asper* (FJ559160) and *G. leucocephalus* (FJ559171), belonging to different subgenera, for outgroup rooting. This alignment contained 86 sequences. We computed matrices of average genetic distance (uncorrected p-distance values transformed into percent, using the pairwise deletion option) within and between individuals belonging to the four *Phylacomantis* taxa. Distances were computed using MEGA, v. 7.0.21 (Kumar et al. 2016) (Table 3).

We conducted Bayesian inference (BI) searches based on 511 bp of the 16S fragment (Fig. 2). The determination of the best-fitting substitution model based on the corrected Akaike information criterion (AICc) was determined in jModelTest2 (Darriba et al. 2012). Phylogenetic analyses were conducted in MRBAYES v. 3.2.6 (Ronquist et al. 2012) on CIPRES Science Gateway v. 3.3 (Miller et al. 2010). The Markov chain Monte Carlo sampling included two runs of four chains each (three heated, one cold) sampled every 10^3^ generations for a total of 10^7^ generations. The first 25% of generations were discarded as burn-in, and 7.5 million trees were retained post burn-in and summed to generate a 50% majority rule consensus tree (Fig. 2).

**Table 3.** Genetic distances. Uncorrected p-distance (transformed into percent) matrix between and within (on the diagonal and in bold) species of the subgenus Phylacomantis, for the analysed 16S fragment.

	*G. corvus*	*G. kintana*	*G. atsingy*	*G. pseudoasper*
***G. corvus***	**0.38**%			
***G. kintana***	9.90%	**0.06**%		
***G. atsingy***	12.45%	10.68%	**0.76**%	
***G. pseudoasper***	14.89%	13.42%	12.40%	**1.36**%

## Results

### Justification for the synonymisation of *G. azzurrae* with *G. corvus*

**Figure 3.**
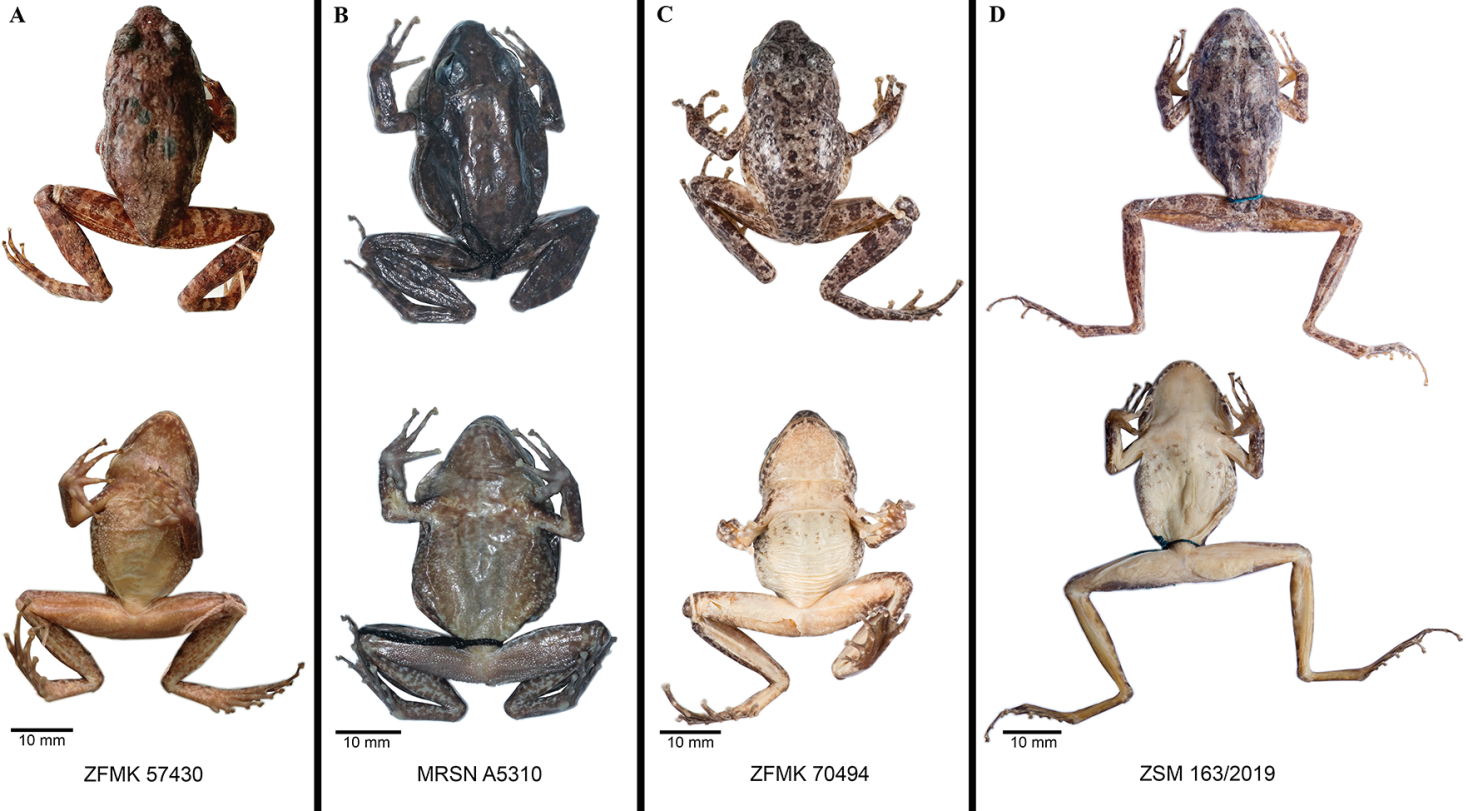
Images of preserved specimens of *Gephyromantis corvus* and *G. kintana* sp. nov. Comparison of dorsal and ventral views of preserved specimens of the two sister species *G. corvus* and *G. kintana* sp. nov. from Isalo, with particular emphasis on the diagnostic ventral colouration. **A** holotype of *G. corvus* (ZFMK 57430), adult male from Namazaha Valley; **B** holotype of *G. azzurrae* (MRSN A5310), adult male from Andriamanero; **C** adult male (ZFMK 70494) used as genetic reference (AF215320) for *G. corvus* in Vences (2000), from an unknown locality of the Isalo Massif (photographs made available by Dennis Rödder and Morris Flecks); **D** holotype of *G. kintana* sp. nov. (ZSM 163/2019, ACZCV_0291), adult male from Zahavola.

The 16S sequence deposited in GenBank with the number AF215320 was obtained from the amplification of a tissue sample of the specimen ZFMK 70494 (Figs 2, 3; Vences 2000). This specimen was collected in Isalo in 1999 when only one *Phylacomantis* species was recognised in the area, and therefore this sequence was associated with the specific name “*corvus*”. No precise locality data are available for the collection of this specimen, but after morphological study of this specimen, and of the type series of *G. corvus* and *G. azzurrae* (Table 2; Figs 2, 3) we conclude that: 1) *G. corvus* and *G. azzurrae* are conspecific (we did not detect any relevant morphological difference between the two holotypes; see following sections for the list of diagnostic characters between the two sympatric *Phylacomantis* taxa from Isalo; see Fig. 3); 2) specimen ZFMK 70494 is different from the holotypes of *G. corvus* and *G. azzurrae* (see below; Fig. 3) and since no name is available for this taxon, it needs to be formally described. ZFMK 70494 and its sequence should no longer be associated with the specific name “*corvus*”.

Mercurio and Andreone (2007) already identified clear morphological differences between the two sympatric species of *Phylacomantis* they found in Isalo (see “Morphological comparison with other species” section in Mercurio and Andreone 2007). Unfortunately, at the time, the authors did not inspect the type specimens of *G. corvus*. The authors detected morphological differences, deep genetic differentiation (> 7.5% p-distance between the two mitochondrial lineages), and bioacoustic differences (based on the comparative analysis of the call of one male from Ambovo and the call of *G. azzurrae* paratype specimen MRSN A5313, now ZSM 0047/2011). Based on these differences they described the taxon *G. azzurrae*. The only *Phylacomantis* sequence available from Isalo at that time was the one obtained from the analysis of the specimen ZFMK 70494. The deep genetic difference they observed between the analysed sequences of the type series of *G. azzurrae* and the sequence of the ZFMK 70494 specimen convinced Mercurio and Andreone (2007) that the lineage they collected in Andriamanero, Iambahatsy, and Sakamalio was different from *G. corvus* and belonged to a different, and still undescribed, species. Although Mercurio and Andreone (2007) were correct in identifying differences in their comparative analyses, their taxonomic conclusions were erroneous. Moreover, the *G. azzurrae* paratype MRSN A5313 (now ZSM 0047/2011) from Sakamalio (KX066634) was not sequenced at the time of the species description. This was done by Cocca et al. (2018), who found it to be conspecific with ZFMK 70494, but not conspecific with the holotype of *G. azzurrae* (MRSN A5310). Cocca et al. (2018) therefore assigned this specimen to *G. corvus* because at this time it was not yet evident that ZFMK 70494 represented an undescribed species.

Based on these observations, we consider *G. azzurrae* as a junior synonym of *G. corvus*. We confirm the existence of two *Phylacomantis* lineages in the Isalo Massif, and we provide the formal description of the unnamed taxon. We follow the integration by the congruence approach proposed by Padial et al. (2010) and define species as independent evolutionary lineages if two or more independent lines of evidence support their distinctness. This new species forms a monophyletic group based on mitochondrial data and differs by an uncorrected pairwise sequence divergence (p-distance) > 7.5% in the analysed 16S fragment from its sister species (Table 3). This value is much higher than the standard value used as threshold for species-level units in amphibians (Fouquet et al. 2007).

We confirmed the distinctness of the two lineages by mitochondrial DNA sequences and morphology, and interpret the concordance between these independent lines of evidence as a strong support their specific distinctness (Avise and Ball 1990).

### Molecular variation and differentiation

The majority rule consensus tree confirms the occurrence of two *Phylacomantis* taxa in Isalo (Fig. 2) and provides evidence for the first unambiguous record of one of these two lineages outside of Isalo (Figs 1, 2). These two lineages are sister taxa (Posterior probability = 100), and together they are the sister group of *G. atsingy* (Posterior probability = 97), which is currently known only from Tsingy de Bemaraha, in western Madagascar. Together, these three *Phylacomantis* species are the sister group of *G. pseudoasper* (Posterior probability = 100), which is the only *Phylacomantis* species found in rainforest habitat and distributed in the north of Madagascar.

The analysed specimens of the two *Phylacomantis* lineages were genetically uniform and showed limited intraspecific divergence (Table 3). All the samples of *Phylacomantis* collected at Anja Reserve, Sakaviro and Tsaranoro cluster together with samples from Sakamalio, Iambahatsy and Tsitorina, and show evidences of slight genetic differentiation with the samples collected at Andriamanero, Canyon des Rats and Namazaha Valley. The second *Phylacomantis* lineage present in Isalo seems to be microendemic to this sandstone massif. The genetic distance observed between the different *Phylacomantis* taxa ranges between 9.9% (comparison between the two *Phylocomantis* taxa occurring in Isalo), and 14.9% (comparison between *G. corvus* and *G. pseudoasper*). More details on 16S genetic distances between species of the Phylacomantis subgenus are provided in Table 3.

### Taxonomy

#### Gephyromantis (Phylacomantis) kintana sp. nov.

Taxon classificationAnimaliaAnuraMantellidae

B5A0191E-DAC4-58AF-B3B9-529391532757

http://zoobank.org/7E684B14-3C30-48E0-8911-2A3D4501EE94

Figures 3D , 4D

##### Etymology.

Mercurio and Andreone (2007) dedicated *G. azzurrae* to F. Andreone’s second daughter, Kintana Azzurra Andreone. Since this name turned out to be a junior synonym of *G. corvus*, F. Andreone and the other authors of this paper wish to dedicate the new species to honour her with the new name. The Malagasy word "*kintana*" means "star" and is used as a noun in apposition.

##### Remarks.

DNA sequences of this species have been wrongly referred to as *Gephyromantis corvus* by Vences (2000; AF215320), Vieites et al. (2009; AF215320), Crottini et al. (2011a; HQ640422–HQ640425), Kaffenberger et al. (2012; JN664348), Cocca et al. (2018; KX066608–KX066637) and all other studies where these accession numbers have been used.

##### Holotype.

**Figure 4.**
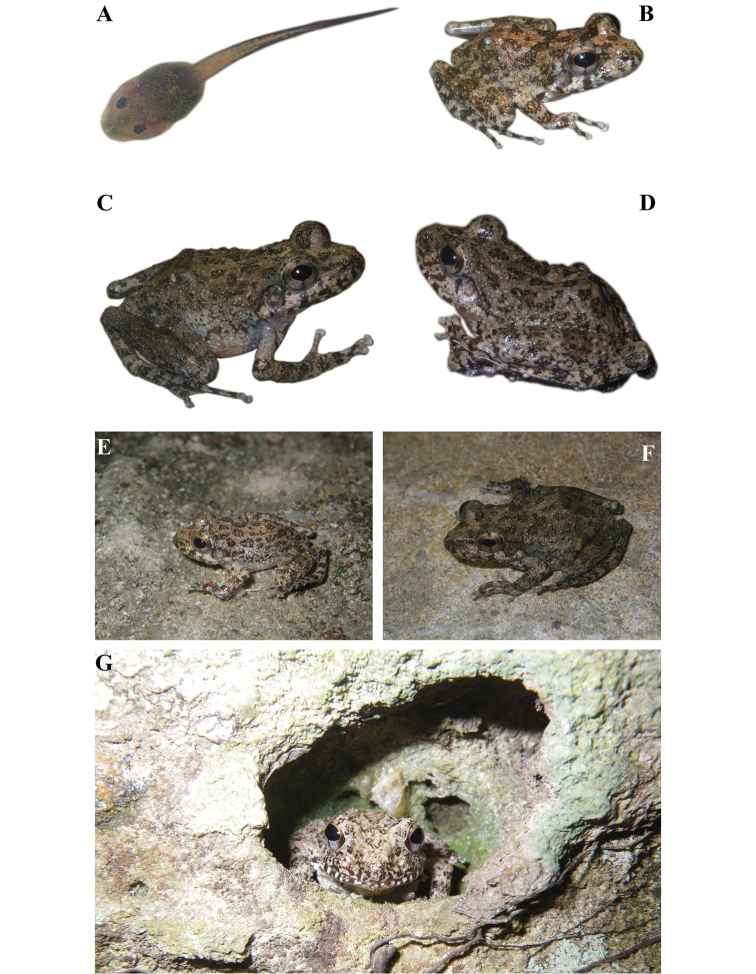
Images of living individuals of *Gephyromantis kintana* sp. nov. Living individuals of *G. kintana* sp. nov. showing the main developmental stages of the newly described species and its natural environment. **A**UADBA-A uncatalogued (ACZCV_0297), tadpole from Malaso, dorsal view; **B** ACZC 6920 (individual not collected), juvenile from Zahavola, dorsolateral view; **C** ACZC 6919 (individual not collected), adult female from Zahavola, dorsolateral view; **D**ZSM 163/2019 (ACZCV_0291), adult male holotype from Zahavola, dorsolateral view; **E** ACZC 6921 (individual not collected), juvenile from Zahavola found on rocks, dorsolateral view; **F** ACZC 6919 (individual not collected), adult female from Zahavola on rocky substrate, dorsolateral view; **G** ACZC 1853 (individual not collected), adult from Malaso hiding in a hole in the wall of the canyon. Photographs by Angelica Crottini.

ZSM 163/2019 (ACZCV_0291; Figs 3D, 4D; tissue sample taken for genetic analysis: MT043942), adult male from Zahavola (Isalo, Ihorombe region, Ranohira Fivondronona, southwestern Madagascar), -22.6215361S, 45.358667E , ca. 881 m a.s.l., canyon with a narrow gallery forest laying on the edge of the border of Isalo National Park, collected on 26 November 2014 by F. Andreone, A. Crottini, and G. M. Rosa.

##### Paratypes.

ZSM 164/2019 (ACZCV_0292; tissue sample taken for genetical analysis: KX066612), adult female, collected at the same locality and date and by the same collectors of the holotype; MRSN A5324 (FAZC 12758; tissue sample taken for genetical analysis: KX066613 and HQ640424), adult male collected at the same locality as the holotype on 17 November 2004 by F. Andreone; ZSM 1553/2009, adult male collected in an imprecise locality within Isalo National Park (original collection data: “Isalo, zwischen Canyons und Piscine” probably referring to a locality close to Piscine Naturelle; Isalo, Ihorombe region, Ranohira Fivondronona, southwestern Madagascar), -22.559667S, 45.371833E, ca. 890 m a.s.l., on 5 June 2003 by N. Lutzmann; ZSM 165/2019 (FAZC 14355; tissue sample taken for genetical analysis: KX066633), adult male, collected at Malaso (Isalo, Ihorombe region, Ranohira Fivondronona, southwestern Madagascar), -22.5885S, 45.35533333E, ca. 966 m a.s.l., a shallow canyon with almost no gallery forest and included within Isalo National Park, on 30 November 2009 by F. Andreone, A. Crottini and G. M. Rosa; MRSN A5322 (FAZC 12627), adult male, collected at Malaso (Isalo, Ihorombe region, Ranohira Fivondronona, southwestern Madagascar), -22.5885S, 45.35533333E, ca. 966 m a.s.l., on 22 November 2004 by F. Andreone; MRSN A5323 (FAZC 12661; tissue sample taken for genetical analysis: KX066635, HQ640422), adult female collected at Malaso (Isalo, Ihorombe region, Ranohira Fivondronona, southwestern Madagascar), -22.5885S, 45.35533333E, ca. 966 m a.s.l., on 24 November 2004 by F. Andreone; MRSN A5373 (FAZC 12859; tissue sample taken for genetical analysis: KX066632, HQ640423), adult male collected at Tsiombivositra (Isalo, Ihorombe region, Ranohira Fivondronona, southwestern Madagascar), -22.3025000S, 45.3583330E, ca. 900 m a.s.l., a locality close to the border but included within Isalo National Park, on 11 December 2004 by F. Andreone; MRSN A5325 (FAZC 13000; tissue sample taken for genetical analysis: KX066631) and MRSN A5326 (FAZC 13001), adult females collected at Ambovo (Isalo, Ihorombe region, Ranohira Fivondronona, southwestern Madagascar), -22.508S, 45.3525E, ca. 999 m a.s.l., within the borders of Isalo National Park, on 18 December 2004 by F. Andreone; MRSN A2786 (FAZC 11964; tissue sample taken for genetical analysis: HQ640425), adult female collected at Andranomena (Isalo, Ihorombe region, Ranohira Fivondronona, southwestern Madagascar), -22.740167S, 45.275E, ca. 740 m a.s.l.), a locality close to Ilakaka and situated outside of the borders of Isalo National Park, on 25 January 2004 by V. Mercurio and J. E. Randrianirina.

##### Diagnosis.

A species assigned to the genus *Gephyromantis* (*sensu*Glaw and Vences 2006), subgenus Phylacomantis, based on genetic and morphological similarities to the other known species (*G. atsingy*, *G. corvus*, and *G. pseudoasper*), and recognisable by the presence of the following morphological characters and natural history traits: (1) medium size (adult male SVL 36–44 mm), (2) webbing between toes present, (3) lateral metatarsalia partly connected, (4) inner and outer metatarsal tubercles present, (5) presence of femoral glands of “Type 2” (*sensu*Glaw et al. 2000), (6) presence of a paired subgular vocal sac, (7) tongue bifid, (8) enlarged triangular finger tips; (9) dirty white throat, belly and thighs, (10) males with white vocal sacs; (11) brownish to olive-grey dorsal colouration with multiple and irregular brown-olive patches, (12) occurrence in young (shallow) canyons with limited (to almost no) vegetation, (13) mostly crepuscular/nocturnal activity, (14) advertisement call (see Mercurio and Andreone 2007 for the description of the advertisement call of specimen MRSN A5313 (ZSM 0047/2011), now genetically assigned to *G. kintana* sp. nov.).

The new species differs from the three other species of *Phylacomantis* by high genetic differentiation (pairwise 16S distance ranging from 9.9% to 13.4%), as well as from a combination of morphological and natural history traits.

*Gephyromantis kintana* sp. nov. is overall similar to the other three species of the subgenus Phylacomantis. Distinguished from *G. pseudoasper* by: (a) dirty white throat (vs. darker colouration); (b) ventrally dirty white thighs (vs. orange colouration); (c) presence of white vocal sacs (vs. blackish vocal sacs); (d) less granular dorsal skin; (e) larger size (maximum SVL in males 43.6 vs. 37.4 mm), (f) higher maximum number of granules in the femoral glands (96 vs. 43), (g) occurrence in young (shallow) canyons with limited vegetation (vs. mostly rainforest), (h) advertisement call (15–21 vs. 3 notes per call and lower dominant frequency, 3,000–3,200 Hz vs. 3,400–5,000 Hz).

Distinguished from the sympatric *G. corvus* by: (a) brownish to olive grey dorsal colouration with multiple and irregular brown-olive patches (vs. darker brown dorsal colouration, often with a broad vertebral stripe), (b) dirty white throat (vs. dark brown throat), (c) dirty white belly (vs. brown belly), (d) dirty white thighs (vs. brown thighs); (e) presence of white vocal sacs (vs. brown-blackish vocal sacs), (f) higher maximum number of granules in the femoral glands (96 vs. 58), (g) occurrence in young (shallow) canyons with limited vegetation (vs. dry deciduous gallery forest in deep canyons), (g) advertisement call (15–21 vs. 10–14 notes per call and higher dominant frequency, 3,000–3,200 Hz vs. 2,400–2,700 Hz).

Distinguished from *G. atsingy* by: (a) brownish to olive grey dorsal colouration with multiple and irregular brown-olive patches (vs. light brown-beige with a greenish shading), (b) less granular dorsal skin; (c) larger size (maximum SVL in males 43.6 vs. 36.6 mm), (d) higher maximum number of granules in the femoral glands (96 vs. 70), e) occurrence in young (shallow) canyons with limited/missing vegetation (vs. “tsingy” geological formations).

##### Description of the holotype

**(Figs 3D, 4D).** Adult male in good state of preservation, distal phalanx of the 5^th^ toe of the left foot removed as tissue sample and part of the ventral surface of thighs cut and opened to count the number of the granules of the femoral gland. SVL 38.2 mm; for other measurements see Table 2. Body slender; head slightly wider than long; snout slightly pointed in dorsal view, rather rounded in lateral view; nostrils directed laterally much nearer to tip of snout than to eye; canthus rostralis moderately defined; tympanum distinct, rounded, its horizontal diameter 0.8 of eye diameter; supratympanic fold well distinct, regularly curved; tongue distinctly bifid posteriorly. Arms slender; subarticular tubercles single; outer metacarpal tubercle poorly developed, inner metacarpal tubercle relatively well developed; fingers without webbing; finger disks triangular and distinctly enlarged; nuptial pads absent. Hind limbs slender; tibiotarsal articulation reaching beyond the snout tip when hindlimbs are depressed along body; lateral metatarsalia partly connected; inner metatarsal tubercle distinct, outer metatarsal tubercle small but recognisable; webbing of foot 1(1), 2i(1.75), 2e(0.75), 3i(2), 3e(1), 4i(2), 4e(2), 5(0.5). Skin slightly granular on dorsum and belly, ventral skin smooth on throat and chest. Femoral glands are distinctly recognisable from external view and are arranged in a typical glandular cluster (“Type 2”, according to Glaw et al. 2000), including 71 single whitish granular glands of ca. 0.3 mm diameter. The vocal sacs in the male holotype are white and distinct.

##### Colouration of the holotype

**(Fig. 3D).** After almost six years in 70% ethanol the holotype conserves the original colour patterns, although it showed a slightly faded dorsal colour (Fig. 3D). Overall grey-brown colouration with distinct darker brown markings. Forelimbs dorsally grey-brown with one distinct darker brown cross-band on upper arm and three brown cross-bands on lower arm and hand, hands speckled. Finger and toe tips are grey-cream and first and second toes are lighter in colour than the other toes. Flanks with the same colour of the dorsum but with less distinct darker patches. Overall the darker dorsal colour fades into the whitish ventral colour. Nostril distinctly surrounded by a cream thin line; lateral head same colour as dorsum. Ventral colouration in the preserved specimen is more contrasted than in the living specimen. Throat, belly and thighs dirty white, ventral shanks slightly darker than thighs, chest flecked with a few distinct and scattered grey-brownish markings; sole of foot brown. Colouration of limbs is overall similar to the dorsum, although it has less defined markings. Hindlimbs with five dark brown cross-bands on femur, four on tibia, four on tarsus and foot; dorsal foot grey-brown with four slightly defined perpendicular darker brown crossbands.

##### Colouration of the holotype in life

**(Fig. 4D).** The live dorsal colouration, based upon photographs, is olive grey with multiple and irregular brown-olive patches with some greenish and orangish shades in dorsal surface. These markings are more contrasted than in the preserved specimen (Figs 3D vs. 4D). The tympanum is cream with multiple small light brown markings. Slightly defined interocular bar. Flanks with multiple brown-olive flecks that become increasingly smaller ventrally. Brown-olive irregular markings present also in lateral head. Hindlimbs dorsally olive grey with brown-olive crossbands and markings and some greenish and orangish shades. Ventral skin colouration in life unknown. The iris of the holotype is golden with a thin black vertical line in the lower portion of the eye, and a mid-horizontal metallic reddish brown broad band.

##### Variations.

Individuals of *G. kintana* sp. nov. have small variations in colouration if compared with the holotype (see Fig. 4B, C, E–G). In life, dorsal colouration can have a very variable number of irregular brown-olive patches. The female paratype ZSM 164/2019 has a lighter dorsum and the dark spots are more contrasted and more visible than in the holotype. The female paratype MRSN A5326 has darker markings on the chest. Two juveniles of *G. kintana* (specimens not collected) showed multiple copper markings on dorsum and on forelimbs and hindlimbs (Fig. 4B, E).

The number of granules composing the femoral glands varies among the analysed specimens. The holotype has 71 granules while specimens MRSN A5322, ZSM 1553/2009, ZSM 0047/2011 (former MRSN A5313) and ZSM 165/2019 have respectively 24, 35, 29 and 75 granules. Paratype MRSN A5373 has the highest number of granules (96; Table 2).

We observed minor variation in the webbing formula in two paratypes, with specimen ZSM 164/2019 with 1(1), 2i(1.75), 2e(0.5), 3i(2), 3e(1), 4i(2), 4e(2.25), 5(0.5); and ZSM 165/2019 with 1(1), 2i(1.5), 2e(0.5), 3i(2), 3e(0.75), 4i(2), 4e(2.25), 5(0.5).

##### Distribution.

**Figure 5.**
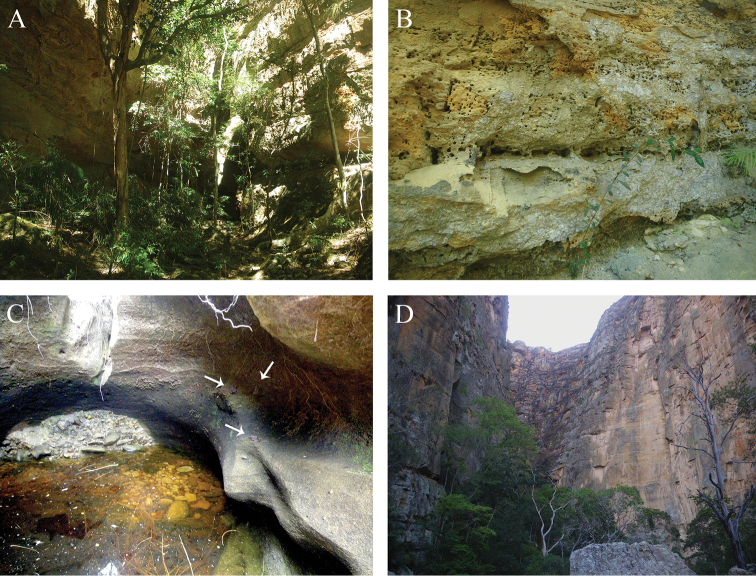
Habitats of *Gephyromantis corvus* and *G. kintana* sp. nov. **A** vegetation within Zahavola Canyon, type locality of *G. kintana* sp. nov.; **B** vertical walls at Zahavola with multiple holes, which are used as shelters by *G. kintana*; **C** lentic water at Malaso Canyon. Multiple *G. kintana* sp. nov. individuals are sitting on the wall of the canyon ca. 1 m above the water (arrows point to the three specimens); **D** Canyon des Rats with its typical dry deciduous gallery forest, habitat of *G. corvus*. Photographs by Angelica Crottini.

*Gephyromantis kintana* sp. nov. is currently known from localities inside (Piscine Naturelle, Zahavola, Sakamalio, Malaso, Tsiombivositra, and Ambovo) and outside (Andranomena, Andranombilahy, Andohasahenina, and possibly Ilakaka) the borders of Isalo National Park (Fig. 1). However, this known distributional area remains restricted to the southern and western portion of the Isalo Massif. Now that we provided a straightforward way to distinguish this species from its sympatric sister species *G. corvus* more field surveys should be conducted to characterise its distribution in detail. The range encompasses elevations from 726–999 m a.s.l. The population densities are not known but it can be locally abundant, with several individuals grouping together close to remaining water bodies forming ponds in shallow (young) canyons (see Fig. 5C).

##### Natural history.

*Gephyromantis kintana* can be found in relatively undisturbed areas in shallow (young) canyons in the Isalo Massif (Fig. 5A–C). Different from the individuals of *G. corvus* in Isalo, that are generally found calling from low vegetation on the lower branches of the trees laying over canyon streams (Fig. 5D), males of *G. kintana* generally call from rocks within the canyon (see Figs 4G, 5C). Most of the sampled individuals were found on the walls of the canyons, and most of the times far from the trees. Different from old (deeper) canyons, which are characterised by the occurrence of a dense gallery forest (Fig. 5D), younger canyons are generally surrounded by sparse vegetation (Fig. 5A). In Malaso, *G. kintana* was often observed sitting on the walls, approximately 1 m above the water (Fig. 5C), while in Zahavola the species was observed using holes on the sandstone walls as shelters (Figs 4G, 5B). Active individuals were found during the day and at dusk, when males start calling quite loudly, but also at night.. The tadpoles of *G. kintana* found at Malaso have a dark fin (Fig. 4A). *Phylacomantis* tadpoles with a reddish fin are also known, but there is no molecular taxonomic identification for this material, and it is therefore not yet possible to conclude if this colour variation (black vs. red) is a diagnostic character between the two *Phylacomantis* species inhabiting the Isalo Massif. Acoustic communication in tadpoles of *G. kintana* has yet to be recorded, which would not be surprising considering the sound repertoire described in *G. corvus* larvae (see Reeve et al. 2011).

##### Conservation status.

If suitable habitat is considered to encompass all areas within the polygon drawn among the known localities (likely an over-estimate), then the EOO (extent of occurrence) totals 563 km^2^. If plots with a scale of 2 km^2^ are used to estimate AOO (area of occupancy), then this species occurs within 36 km^2^ of habitat. Based on IUCN Red List guidelines (IUCN Standards and Petitions Subcommittee 2019) we propose that *G. kintana* should be considered as Endangered (under criterium B1ab(iii)+2ab(iii)). This suggestion considers the species’ narrow distribution and apparent restriction to inhabit young canyons as well as the fact that the area of the Isalo Massif not included in the borders of the National Park is under severe exploitation by various anthropogenic activities (Mercurio et al. 2008). In these areas the main threats are: 1) the use of periodic, and often uncontrolled fires to maintain the savannahs (Mercurio et al. 2008); 2) excavations for sapphire mines (Duffy 2006); and 3) unsustainable logging of remaining gallery forests (Mercurio et al. 2008). Despite very limited information on this species, the ongoing pressure on the extent and quality of the habitat is expected to impact the populations likely leading to their declines. Rakotondravony and Goodman (2011) listed *G. corvus* for the Makay Massif region (also discussed in Cocca et al. 2018). We do not consider this record here because we did not have access to any voucher material from this area at this time, and from the available photographs it was not possible to unequivocally assign this geographic record to either *G. corvus* or *G. kintana*.

## Discussion

In this study we synonymised *G. azzurrae* with *G. corvus* based on molecular and morphological evidence, and described *G. kintana* sp. nov., which, as far as we know, represents a new microendemic species of the Isalo Massif. The most recent species account for the area reported the occurrence of 47 reptile taxa and 24 amphibians (Cocca et al. 2018). In this recent paper, five amphibian taxa were considered Isalo endemics: *Gephyromantis azzurrae*, *Mantella expectata*, *Scaphiophryne gottlebei*, *Mantidactylus noralottae*, and M. sp. aff. multiplicatus Ca65 “Isalo” (Cocca et al. 2018). Now, despite the synonymisation of *G. azzurrae* with *G. corvus*, the number of endemic amphibian taxa of the Isalo Massif remains the same since we could add *G. kintana* to this list.

The inspection of the ventral colouration is the clearest morphological difference between this pair of sympatric sister species and represents the most straightforward way to discriminate these taxa in the field. This morphological variation has been known for several years, but for a long time it was thought to reflect individual variation within the same species. Disentangling this situation was possible only after the inspection of all photographic records, of specimen ZFMK 70494 (the individual from which the first *Phylacomantis* sequence from Isalo has become available) and of all specimens formerly assigned to *G. corvus* and *G. azzurrae* in the herpetological collections of MRSN and ZSM, in combination with the compilation of all genetic data. This approach enabled us to conclude that specimen ZFMK 70494 was different from the holotypes of both *G. corvus* and *G. azzurrae*, that ventral colouration represented a diagnostic character and that the second *Phylacomantis* species still needed to be formally described (Fig. 2). Based on these findings, we also clarify that the tadpoles studied in Reeve et al. (2011) and assigned to *G. azzurrae* actually belong to *G. corvus* and, so far, the sound emission has not been observed in the tadpoles of *G. kintana* sp. nov.

**Figure 6.**
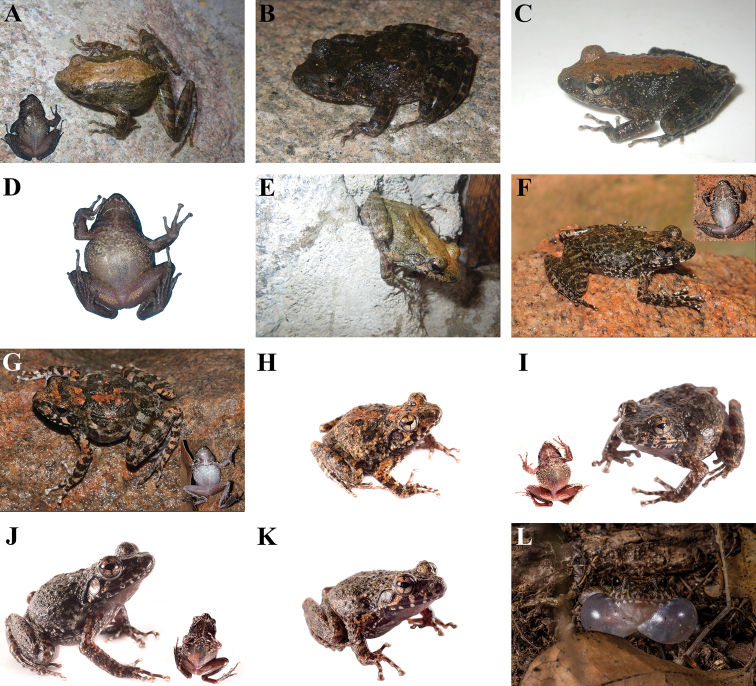
Images of living individuals of *G. corvus*. Photographs showing dorsal and ventral colour variability and habitats of *G. corvus*. When both ventral and dorsal picture of one individual are available, ventral colouration is given in inset. **A**ZSM 156/2019 (ACZCV_0288) adult male from Antsifotra Canyon; **B**ZSM 154/2019 (ACZCV_0286) adult female from Antsifotra Canyon; **C** ACZCV_0290 (UADBA-A uncatalogued) adult female from Antsifotra Canyon; **D**ZSM 155/2019 (ACZCV_0287) ventral view of an adult male from Antsifotra Canyon; **E** ACZCV_0289 (UADBA-A Uncatalogued) adult male from Antsifotra Canyon; **F**ZSM 159/2019 (FAZC 15812) adult female from Anja Reserve; **G**ZSM 160/2019 (FAZC 15813) adult male from Anja Reserve; **H** ACZC 10901 (individual not collected) adult male from Sakaviro; **I** ACZC 10904 (UADBA-A uncatalogued) adult male from Sakaviro; **J** ACZC 10957 (individual not collected) adult male from Tsaranoro; **K** ACZC 10958 (UADBA-A uncatalogued) adult male from Tsaranoro; **L** ACZC 10964 (individual not collected) adult male from Tsaranoro calling from the leaf litter. Photographs **A–E** by Angelica Crottini, **F–G** by Franco Andreone, **H–L** by Javier Lobón-Rovira.

This study provid evidence for the occurrence of *G. corvus* ca. 200 km away from the Isalo Massif (Figs 1, 6). This finding, together with the synonymisation of *G. azzurrae* with *G. corvus*, may likely require the modification of the IUCN Red List assessment of *G. corvus*, highlighting how important it is to combine the efforts of performing herpetological surveys with the molecular characterisation of the collected material. Although demanding, this combined method is proving to be an excellent way to increase our knowledge on species distribution and is boosting our capacity to discover candidate species and update our reference molecular databases. Since 2004, 140 new species of amphibians have been described from Madagascar (AmphibiaWeb 2020), but the number of candidate species is still very high (Vieites et al. 2009; Perl et al. 2014; but see also Glaw et al. 2019 for a more updated estimate of the candidate species and their formal description). In the last 15 years ten major taxonomic revisions on the amphibians of Madagascar have been published (Glaw and Vences 2006; Glaw et al. 2010; Vences et al. 2010; Köhler et al. 2010, 2015; Wollenberg et al. 2012; Rakotoarison et al. 2015, 2017; Scherz et al. 2017, 2019), all of which represented crucial steps in unveiling the biodiversity value of this irreplaceable hotspot of biodiversity. The currently discussed case demonstrated how vouchering continues to be an extremely valuable tool that enable specimen direct comparison and facilitate the identification, and taxonomic revision of collected material and name bearing types (Funk et al. 2005; Rocha et al. 2014).

## Supplementary Material

XML Treatment for Gephyromantis (Phylacomantis) kintana
